# Hands‐on protocol for preparing water‐soluble fractions from agri‐food samples for NMR‐based metabolomics analysis

**DOI:** 10.1002/2211-5463.70153

**Published:** 2025-10-31

**Authors:** Andrea Fernández‐Veloso, Jaime Hiniesta‐Valero, Alejandra Guerra‐Castellano, Miguel A. De la Rosa, Irene Díaz‐Moreno

**Affiliations:** ^1^ Instituto de Investigaciones Químicas (IIQ), Centro de Investigaciones Científicas Isla de la Cartuja (cicCartuja) Universidad de Sevilla–Consejo Superior de Investigaciones Científicas (CSIC) Spain; ^2^ ManSciTech S.L. Sevilla Spain

**Keywords:** agri‐food, metabolomics, nuclear magnetic resonance, omic data

## Abstract

The aim of this study was to address the lack of protocols for nuclear magnetic resonance (NMR)‐based metabolomics in the agri‐food sector by providing a reproducible workflow for the preparation and analysis of water‐soluble metabolite fractions. These fractions, rich in primary metabolites such as sugars, amino acids, and organic acids, are key to assessing agri‐food products' composition, quality, as well as to monitor their manufacturing and development processes. The protocol differentiates solid and liquid matrices, optimizing extraction procedures accordingly. Representative agri‐food products—strawberry leaves (solid) and wine (liquid)—were analyzed to demonstrate the method's versatility and applicability. Key steps include tailored sample preparation, optimization of NMR acquisition, and spectral quality control, ensuring high data quality and reproducibility. The proposed workflow enhances reproducibility across agri‐food metabolomics studies and facilitates integration into broader food quality, traceability, and safety frameworks.

## Graphical abstract

This research protocol outlines a workflow for nuclear magnetic resonance (NMR)‐based metabolomics in the agri‐food sector. Using two case studies—strawberry leaves (solid matrix) and wine (liquid matrix)—it details the procedures for sample preparation, data acquisition, and processing.

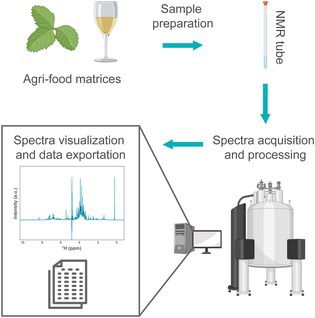

**Abbreviations**
CDCl_3_
: deuterated chloroform
D1: relaxation delay
D_2_O: deuterium oxide
DS: dummy scans
EtOH: Ethanol
FID: free induction decay
FT: fourier transform
MS: mass spectrometry
NMR: nuclear magnetic resonance
NS: number of scans
O1: transmitter offset frequency
P1: pulse length
QR: quality reference
RG: receiver gain
SW: spectral width
TD: size of the FID
TMS: Tetramethylsilane
TSP: 3‐(TrimethylSylil) Propionic‐2,2,3,3‐d4 acid

Metabolomics is a powerful omics discipline dedicated to the comprehensive analysis of metabolites—the small‐molecule end products of cellular processes—in biological systems. Unlike nucleic acids or proteins, metabolites provide a direct snapshot of an organism's physiological and biochemical state, making metabolomics particularly valuable for functional phenotyping and system‐level understanding.

The most widely employed analytical platforms in metabolomics are mass spectrometry (MS) coupled with gas chromatography (GC) or liquid chromatography (LC), and nuclear magnetic resonance (NMR) spectroscopy. Among these, MS remains the most commonly used due to its high sensitivity and extensive metabolite coverage. Nevertheless, NMR spectroscopy offers unique advantages, including excellent reproducibility, minimal sample preparation, nondestructive analysis, and the ability to detect a broad range of polar compounds without the need for derivatization [1, 2].

While metabolomics has traditionally focused on human health and disease [3], its applications have rapidly expanded into fields such as toxicology, nutrition, environmental science, and notably, the agri‐food sector. In particular, NMR‐based metabolomics has gained traction in agri‐food research over the past decade [4], given this sector's importance in public health, economic development, and environmental sustainability [5].

However, despite the growing body of work in this area, the lack of published protocols for NMR‐based metabolomics in agri‐food applications remains a significant barrier [6]. Methodological variability affects data quality, hinders reproducibility, and complicates cross‐study comparisons, ultimately limiting the integration of metabolomic data into shared databases and industrial workflows.

This issue is not unique to agri‐food research. Across the broader metabolomics field, methodological standardization is a persistent challenge. Variability in sample preparation, instrument settings, and data processing can lead to inconsistent results, making harmonized protocols essential for generating robust, comparable, and high‐quality datasets. Addressing this need is particularly urgent in applied fields such as food science, where metabolomics has the potential to transform quality control, traceability, and authentication practices.

In this context, NMR metabolomics is a suitable tool for food quality control, safety, traceability (especially for products with Protected Designation of Origin), and on‐site monitoring [7]. Yet, its broader adoption is hindered by the lack of harmonized workflows tailored to the specific characteristics of agri‐food matrices. Although various studies have employed NMR‐based metabolomics in this sector, few have provided validated and broadly applicable protocols [4].

To address this gap, we present a reproducible protocol for the preparation and NMR analysis of water‐soluble metabolite fractions from agri‐food products. This fraction includes key primary metabolites—such as sugars, amino acids, and organic acids—that are critical for profiling food composition and assessing quality attributes [8]. The protocol accounts for the nature of the sample by distinguishing between liquid and solid matrices, ensuring optimized handling and extraction procedures for each type.

Wine and strawberry leaves analyzed in the context of product authentication and crop monitoring through metabolic state studies, respectively, are herein used to illustrate the protocol's application to both liquid and solid agri‐food matrices [9, 10]. The workflow also incorporates essential elements of the metabolomic pipeline, including experimental design, optimization of NMR acquisition parameters, and spectral quality control. These considerations are critical for enhancing reproducibility and enabling reliable batch acquisition and data integration (Fig. 1).

**Fig. 1:**
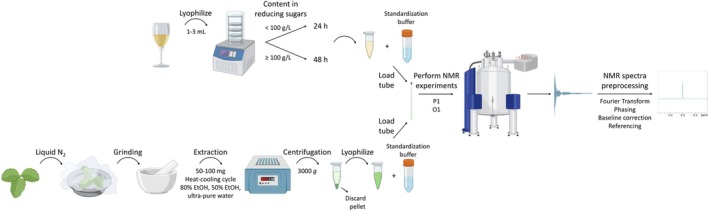
Schematic overview of the NMR‐based metabolomic workflow for water‐soluble fractions from liquid and solid agri‐food samples. The initial sample preparation steps differ depending on the physical state of the sample; however, both protocols converge after lyophilizate resuspension. Subsequent steps—NMR parameter optimization, batch of spectra acquisition, and transformation to omics format—are similarly performed independently of the nature of the product. O1, transmitter frequency offset; P1, pulse length.

By providing a practical and adaptable framework, this protocol aims to facilitate robust metabolomic profiling across diverse agri‐food matrices. In doing so, it contributes to ongoing efforts toward methodological harmonization and supports the broader implementation of NMR‐based metabolomics in food science and industry.

## Materials

### Water‐soluble fraction extraction

1. Liquid Nitrogen
2. Mortar and pestle
3. Precision scale
4. Spatula
5. 1.5 mL polypropylene microcentrifuge tubes
6. 80% ethanol (EtOH)
7. 50% EtOH
8. Ultra‐pure water
9. Block heater
10. Box with ice
11. Centrifuge
12. 5‐mL polypropylene tubes with caps
13. 1‐mL graduated micropipette
14. Pipette tips

### NMR sample preparation and spectra acquisition

15 Needle
16 Aluminum foil
17 Liquid Nitrogen
18 Freeze dryer
19 1.5 mL polypropylene microcentrifuge tubes
20 Deuterium oxide (D_2_O) 99.9%
21 Distilled water
22 Phosphate buffer pH 7: 0.2 m NaH_2_PO_4_, 5 mm 3‐(TrimethylSylil) Propionic‐2,2,3,3‐d4 acid (TSP) (97%) and 30 mm NaN_3_. Store at room temperature
23 Oxalate buffer pH 4: 400 mm oxalate buffer (pH 4.0), 5 mm TSP (97%). Store at room temperature in the dark
24 1 mL and 200 μL graduated micropipettes
25 Pipette tips
26 Centrifuge
27 Glass Pasteur pipettes
28 Ultrasonic bath
29 5 mm NMR tubes
30 NMR spectrometer equipped with autosampler (required for automated acquisition in batches, see steps #**15–18** from Section Parameter optimization to perform Nuclear Magnetic Resonance experiments on automated batches)

### Software

1. RStudio (1.4.1717) [11] (required for randomization, see steps #**2–3** from Section Experimental design randomization and quality references).
2. TopSpin 4.3.0 (Bruker BioSpin GmbH, Rheinstetten, Germany).
3. IconNMR Automation Software for TopSpin 4.3.0 (Bruker BioSpin GmbH, Rheinstetten, Germany) (required for automated acquisition in batches, see steps #**15–18** from Section Parameter optimization to perform Nuclear Magnetic Resonance experiments on automated batches).

## Methods

### Experimental design randomization and quality references

1. Generate a file that contains the names for each of your samples.
2. For experimental design randomization, load the file as a variable in *RStudio* with the names of your samples.
3. Randomize the order of the names using the *sample()* function and save the output as a new variable; this will define the order in which you should prepare the samples and acquire the spectra. More information in **Tips and Tricks/Troubleshooting** #**1**.
4. Group the samples into batches based on the number of samples you can acquire in a single day according to your autosampler capacity, availability of equipment, etc.
5. At a minimum, the first and last sample on each batch must be a quality reference (QR) sample. QR sample consists of a mixture of all the samples in the batch, thus representing the variability among the group. These samples fulfill two relevant functions. First, they help us optimize the parameters for NMR acquisition, as they represent the cohort of samples (see the Parameter optimization to perform Nuclear Magnetic Resonance experiments on automated batches Section). Second, they also allow us to evaluate whether any systematic effect is being introduced throughout the spectrometer acquisition. This is why it is important to include them, at least, at the beginning and ending of the batch. If a high number of samples are acquired, it is highly recommended that QR samples also be included in the middle of the dataset.

### Water‐soluble fraction extraction from solid matrices

1. Collect the solid material—see **Tips and Tricks/Troubleshooting #2**. Place the strawberry leaves in a mortar. Then, add liquid nitrogen to rapidly freeze the sample and grind it thoroughly until obtaining a fine powder. Measure 100 mg of the ground leaf material using a precision scale and transfer it to a prelabeled 1.5‐mL microtube tube [10].
2. Add 1 mL of 80% EtOH, keep the tube on the block heater at 80 °C for 15 min, and then transfer it to an ice box for 3 min (see **Tips and Tricks/Troubleshooting #3**).
3. Centrifuge the extract at 3000 ***g***, at room temperature for 3 min. Collect the supernatant carefully and transfer it to a prelabeled 5‐mL tube, avoiding the aspiration of the pellet.
4. Repeat step #**2** on the remaining pellet using 50% EtOH instead of 80%; and repeat step #**3** collecting the supernatant in the same prelabeled 5‐mL tube.
5. Repeat step #**2** on the remaining pellet using ultra‐pure water instead of 80% EtOH, and repeat step #**3** collecting the supernatant in the same prelabeled 5‐mL tube. Discard the pellet.
6. Expose the collected supernatant to a nitrogen flux for 20 min to ease ethanol evaporation (see **Tips and Tricks/Troubleshooting** #**4**). Storage information can be found in **Tips and Tricks/Troubleshooting** #**5**.

### Sample preparation for liquid NMR spectroscopy

1. Pipette 1 mL from the liquid aqueous sample into a prelabeled 5‐mL tube (see **Tips and Tricks/Troubleshooting** #**6**). For solid samples, subjected to previous extraction (see the Water‐soluble fraction extraction from solid matrices Section), proceed with the remaining volume after the nitrogen flux **step #6**.
2. Remove the cap from the 5‐mL tube and seal the opening tightly with aluminum foil. Then, pierce the foil using a needle to create five evenly spaced holes.
3. Freeze the sample in liquid nitrogen prior to lyophilization.
4. Lyophilize the sample in a freeze dryer for 24 h if its content in reducing sugars is below 100 g/L; or 48 h if its content is higher. See **Tips and Tricks/Troubleshooting #7** for further information.
5. Resuspend the lyophilized material in 600 μL of standardization buffer composed of 540 μL of D_2_O (99.9%) and 60 μL of phosphate buffer for solid samples; or 400 μL of D_2_O (99.9%) and 200 μL of oxalate buffer for liquid samples [11] (See **Tips and Tricks/Troubleshooting** #**8**).
6. Centrifuge at 7000 ***g***, at room temperature for 10 min and transfer the supernatant with a glass Pasteur pipette to a 5 mm NMR tube.
7. Place the NMR tube in the ultrasonic bath for 5 s to eliminate any air bubbles that may form during the preparation.

### Parameter optimization to perform nuclear magnetic resonance experiments on automated batches

**Fig. 2:**
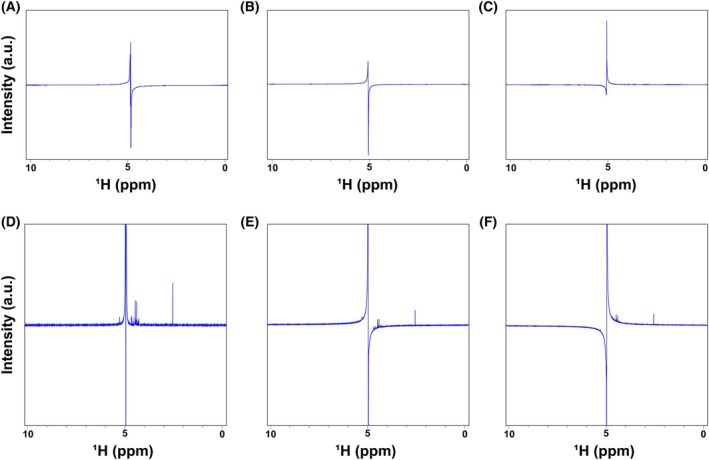
NMR spectra throughout pulse length calibration and presaturation frequency optimization. (A) Appearance of NMR spectrum after applying a 360° pulse without presaturation, in which the solvent signal predominates with respect to the rest of the sample, and presents horizontal symmetry in terms of intensity. (B) Appearance of NMR spectrum after applying a pulse shorter than 360°, in which the negative part of the solvent signal presents higher intensity in absolute value. (C) Appearance of NMR spectrum after applying a pulse longer than 360°, in which the positive part of the solvent signal presents higher intensity in absolute value. (D) Appearance of NMR spectrum after applying a solvent presaturation pulse in the center of the solvent signal, in which the baseline both upfield and downfield the solvent signal is flat. (E) Appearance of NMR spectrum after applying a solvent presaturation pulse at a frequency lower than the center of the solvent signal, resulting in a non‐flat baseline, with upfield signals appearing at lower intensities than those downfield. (F) Appearance of NMR spectrum after applying a solvent presaturation pulse in a frequency higher than the center of the solvent signal, resulting in a nonflat baseline, with downfield signals appearing at higher intensities than those upfield.

1. Introduce the QR sample in the spectrometer and generate a new experiment with the default parameters for a standard 1D‐^1^H 90° pulse experiment (see **Tips and Tricks/Troubleshooting #9**), which can be found as a parameter file called PROTON. These parameters include 16 NS (Number of Scans), 2 DS (Dummy Scans), D1 (relaxation delay) of 1 s, 32 RG (Receiver Gain), 20 ppm of SW (Spectral Width) approximately, and a TD (Size of the Free Induction Decay (FID)) of 65 536. Change the pulse program (*pulprog*) to zg.
2. Set the spectrometer temperature at 298 K.
*te 298*
*teset*
*teready 300 0.1*
3. Set the spectrometer to the correct frequency for proton, lock for your solvent (H_2_O + 10% D_2_O in this case) and shim (See **Tips and Tricks/Troubleshooting** #**10**). Then set the receiver gain to 0.5 and adjust the pulse length (P1) to one‐tenth of the default value provided.
4. Run the first spectrum with 1 NS and 0 DS. Apply the Fourier Transform (FT) to the acquired FID and adjust the zero‐order phase to optimize the parameters.
5. Define the transmitter offset frequency (O1) at the center of the solvent peak from that spectrum, then run the experiment again applying FT to the FID and adjusting the zero‐order phase to check that the solvent peak is the center of the obtained spectrum.
6. To apply an exact 90° pulse to excite the sample, the length value of the pulse (P1) related to the power is not standard for every case; it depends on the properties of the sample itself, including the solvent, and thus needs to be optimized. To optimize P1, first, a theoretical 360° pulse is applied that will eventually be divided by four. To apply a theoretical 360° pulse set the D1 to 10 s and multiply the default P1 (optimized for a 90° pulse) by 4. Run the experiment and process it without further phase adjustment.
7. Check the solvent peak:
  7.1 If the solvent signal presents horizontal symmetry in terms of intensity to both positive and negative values, it means the P1 applied is exactly a 360° pulse (Fig. 2A).
  7.2 If the solvent signal does not present horizontal symmetry, being more intense for negative values, it means that the length of the pulse was too short, and thus the applied P1 is lower than 360° (Fig. 2B). In this case, the P1 value should be increased until a symmetrical signal (**step** #**7.1**) is obtained.
  7.3 If the solvent signal does not present horizontal symmetry, being more intense for positive values, it means that the length of the pulse was too long, and thus the applied P1 is higher than 360° (Fig. 2C). In this case, the P1 value should be decreased until a symmetrical signal (**step** #**7.1**) is obtained.

It is important to note that every time the P1 value is changed, it is necessary to run the experiment again and process it without adjusting the phase to check the solvent signal.

8 After adjusting the P1 value according to **step** #**7** to obtain a real 360° pulse, the P1 value should be divided by 4, to obtain a real 90° optimized for the samples. The obtained P1 value for an exact 90° pulse may slightly vary across different batches.
9 The spectrum is dominated by the solvent peak (water); therefore, presaturation is applied to reduce its intensity and better observe metabolite signals. Create a new experiment with the zgpr pulse program, set the presaturation O1 to the solvent peak center, and load the parameters.
10 Define O1 to the center of the solvent signal again in this new experiment and set P1 value to apply an exact 90° pulse (see **steps** #**6–8**). As the water signal is presaturated, it is necessary to determine the optimal value of the RG with the command *rga*, which is expected to remain constant throughout the several batches of samples of similar nature acquired in the same conditions (including equipment, probe, etc.). If the receiver gain value changes under the same conditions, force the one obtained from the first batch. In the samples used as examples, the RG used was 36 for strawberry leaves extracts, and 101 for wine samples.
11 Run the experiment and apply the FT to the FID. Setting the pivot point at the center of the internal reference signal (TSP for water‐soluble samples), manually adjust the zero‐order phase until the TSP peak shows a proper phase.
12 Check the solvent signal surrounding regions and, based on the baseline, proceed as follows:
  12.1 If both up‐ and downfield regions across the solvent signal exhibit vertical symmetry, in terms of baseline flattening, then the presaturation pulse was applied to the exact center of the solvent signal, and the presaturation pulse frequency, O1, is correct (Fig. 2D).
  12.2 If the region downfield of the solvent peak shows a baseline higher than the upfield region, the presaturation pulse was applied to lower frequencies of the solvent signal center, and thus the O1 value needs to be increased (Fig. 2E).
  12.3 If the region upfield of the solvent peak shows a baseline higher than the downfield region, the presaturation pulse was applied to upper frequencies of the solvent signal center, and thus the O1 value needs to be decreased (Fig. 2F).

It is important to note that every time the O1 value is changed for optimization, the experiment needs to be acquired and preprocessed again following **step** #**11** before checking the region surrounding the solvent signal.

13 Once the P1 and O1 values are optimized, optimize a final parameter from the NMR experiment for metabolomic profiling of water‐soluble fractions which is the presaturation pulse power. Create a new experiment with the pulse program noesygppr1d/noesygppr1d.2 and change the default P0 and P1 values to the optimized 90° pulse value. Do the same for the default O1 and RG values. Regarding the rest of the acquisition parameters, they can be set as follows: 64 NS, 4 DS, 4 s for D1, 20 ppm for the SW, and 65 536 for the TD.
14 Calculate the power in decibels (dB) for a presaturation pulse of 25 Hz. Optimize the presaturation power to 25 Hz to avoid the suppression of nearby signals adjacent to the solvent signal. A 25 Hz presaturation pulse is recommended to avoid perturbing metabolite signals close to the water region that may be presaturated too when stronger powers are used.
*pulse 25 Hz*

As a result of the previous command, TopSpin indicates the correspondent value in dB for the presaturation pulse. Introduce that value in the power level in dB command.

pldb9 *value*

Up to this point, the parametrization of the experiments has been performed. To perform the acquisition of the whole batch of samples in an automated manner, two requirements need to be fulfilled: the availability of an NMR spectrometer with an autosampler and a console with IconNMR Automation Software [11] installed.

15 Load the samples into the numbered holders of the autosampler following the randomized order.
16 Open the IconNMR Automation Software [11]. It is possible to do it by running the command *icona* in the TopSpin [10] command line.
17 The IconNMR Automation Software [11] interface shows a line for each holder, in which the following information for each sample needs to be filled (See **Tips and Tricks/Troubleshooting** #**11 and** #**12**):

- Name of the sample
- Number of experiment, the expno according to the Bruker directory structure
- Solvent used, in this case, H_2_O + D_2_O
- The name of the NMR experiment to perform
- The set of parameters, including P0, P1, O1, and pldb9, for which the optimized values should be entered
- Title/Orig, to determine the title of the sample and activate automated transference of files to desired destination
- Time duration of the experiment
- User
- Starting time of the experiment

18 After loading the information on each sample in its corresponding line according to the holder, select all the samples, submit the experiments, and start the acquisition.

### Nuclear magnetic resonance spectra preprocessing

1. When using IconNMR Automation Software [11], default spectrum preprocessing is performed according to the Processing AU program (AUNMP) proc_1d, included by default in Topspin. This processing includes FT, chemical referencing and phase and baseline correction. Alternatively, in TopSpin, it is possible to perform these same steps in an automated mode, using the following commands:

- Apply FT to the FID with exponential multiplication line broadening

*ef*

- Adjust both zero‐order and first order phases automatically

*apk*

- Perform chemical shift referencing

*sref*

- Correct the baseline

*abs*

Following acquisition, it is relevant to highlight that subsequent steps which do not require the use of the spectrometer, that is, *Nuclear Magnetic Resonance spectra preprocessing* and *Converting spectral information into omics data format for posterior analysis*, can also be performed in alternative software programs (see **Tips and Tricks/Troubleshooting #13**).

**Fig. 3:**
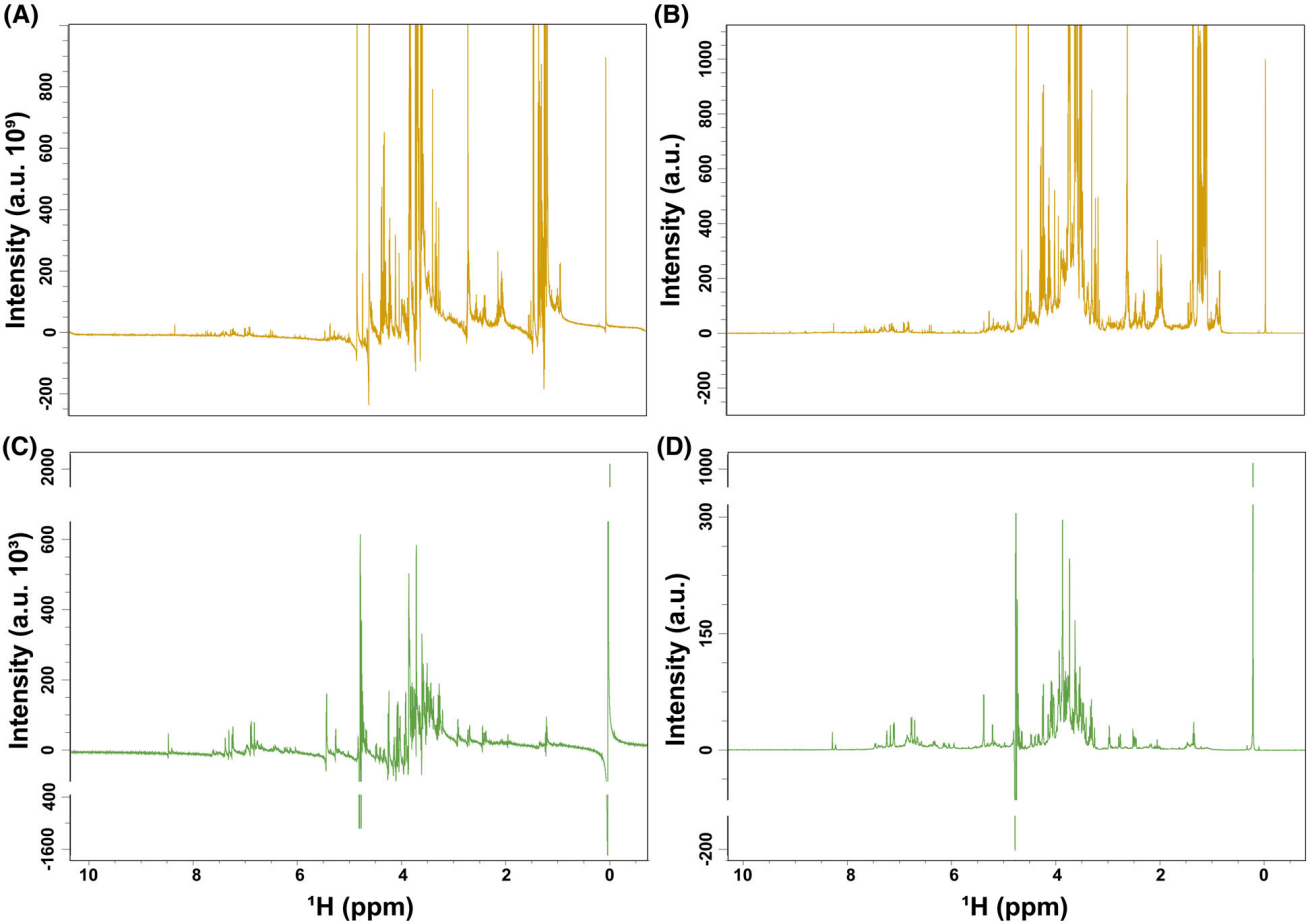
Representative ^1^H‐NOESY spectra from liquid and solid agri‐food samples. (A) Raw spectrum of a liquid sample (*Fino* wine). Pulse sequence: noesygppr1d.2 (B) Processed spectrum of the same liquid sample (*Fino* wine) after automatic phasing, baseline correction and scaling according to the internal standard (TSP; 1 H, 0.00 ppm). (C) Raw spectrum of a solid sample extract (strawberry leaves). Pulse sequence: noesygppr1d (D) Processed spectrum of the same solid sample extract (strawberry leaves) after automatic phasing, baseline correction and scaling according to the internal standard (TSP; 1 H, 0.00 ppm).

2 If following automatic processing, spectra still show imperfections, further optimizations can be made. Adjust manually the phase and/or baseline in the “Process” section of the TopSpin software [10]; usually, adjusting zero‐order phase should be enough to obtain a good spectrum acquired with these instructions. For manual chemical referencing to the internal standard, use the “Calib. Axis” option in this section, set the TSP chemical shift to 0.
3 To dampen small variations in the spectra due to batch acquisition, apply a spectral scaling based on the TSP signal. For this purpose, execute *normCY* command, which is a function from a script provided by Bruker, to set the TSP signal intensity to 1000 (Fig. 3).

### Quality control of acquired nuclear magnetic resonance spectra resolution

1. Display the NMR spectrum in a new window in TopSpin.
2. Focus on the internal reference (TSP) peak by zooming in on that area so that only the TSP peak is visible on screen.
3. Select the TSP's peak with the command *dpl*.
4. Calculate the full width at half height for that peak with the command *hwcal*.
5. Check the line broadening parameter with the command *lb*.
6. For spectra processed with a line broadening of 0.3 Hz, the full width at half the height of the TSP peak should be less than 0.8 Hz to be considered of good quality. When using a line broadening of 1 Hz, the full width at half height should be less than 2.5 Hz to meet quality criteria [12]. It is recommended to verify this throughout parameter optimization using the QR sample, before acquiring the whole batch. Once the QR sample meets the criteria and the whole batch is acquired, if any of the spectra do not meet the criteria, it is recommended to repeat the measurement optimizing the shims for that sample.

### Converting spectral information into omics data format for posterior analysis

1. To combine all spectra from a directory into a single csv file for posterior analysis, first, execute the *multicmd* command.
2. Define the number of spectra in the directory.
3. Introduce the command *xpy getpoints x y*. X is the chemical‐shift value in ppm for the left limit of your spectra; this must be big enough to capture all the signals from your spectra. Y is the chemical‐shift value in ppm for the right limit of your spectra; normally no signals can be found to the right of the TSP signal, so −0.1 is enough. This function is also included in a script provided by Bruker.
4. Execute the script. This will generate two files for each spectrum in its pdata folder: rpoint and chemshift. The first one contains the intensities of each datapoint, and the second one contains the chemical shift values in ppm.
5. To combine the information of the different spectra in one single file, display the first spectrum in a TopSpin window and execute the command *prep_analysis*. Fill “First” with the number of your first experiment and “Last” with the number of the last experiment you want to export. Click OK.
6. Two new files will be created in the folder of the first experiment exported: intensities.csv and datapoints.csv. The intensities.csv file contains the intensity values for each sample as rows. The datapoints.csv file is similar, only that the first row contains the chemical shift values, followed by the intensity information of spectra in the rest of the rows. This file is usually used as input for further analysis.

## Tips & tricks/troubleshooting

1. In multiclass experimental design, block randomization [13] that mixes the different classes in random order allows monitoring for deviations during sample preparation and/or acquisition.
2. It is important to note that the procedure described for solid samples may vary for other agri‐food samples, for instance, alternative plant extracts, including fruits, stems, or roots, especially if their content in nonwater‐soluble compounds is high.
3. The extraction procedure removes ethanol‐soluble components before analyzing the water‐soluble fraction. Some metabolites may dissolve in ethanol but not in water, and thus could be partially lost during resuspension in the NMR buffer. Despite this limitation, ethanol precipitation is necessary to remove macromolecules (e.g., proteins, polysaccharides) that would broaden NMR signals and alter the baseline. This approach provides a reproducible method for the hydrophilic fraction of various agri‐food products ensuring good spectral resolution for small polar metabolites.
4. During ethanol evaporation under nitrogen flux, carefully regulate the current pressure of the nitrogen flux. Too strong currents can cause the sample to spill out.
5. If the entire sample preparation process cannot be completed consecutively, it is important to store the samples below −20 °C to preserve their integrity and prevent degradation.
6. Although the sample volume is less than 5 mL, 5‐mL tubes are recommended for lyophilization due to the presence of ethanol, which can induce the sample to leak out during the drying process if smaller containers are used.
7. The sugar content of an agri‐food product strongly affects freeze‐drying behavior. High sugar concentration increases the viscosity and lowers the glass transition temperature of the frozen matrix, which slows down sublimation and prolongs drying times. In general, sugar content can be determined by standard methods such as refractometry (°Brix), enzymatic or chromatographic assays (HPLC), or inferred from product classification (e.g., sweet vs. dry fruit juices, wines, or purees). Awareness of the sugar level is therefore important when planning freeze‐drying conditions.
8. It is relevant to consider that the chosen approach for sample preparation, especially solvents and buffers used, is highly dependent on the nature of the sample. For instance, in these cases, the solid and liquid samples differ in their pH, since leaves are organs from plants, whose pH is mainly neutral, while wine is a fermented beverage made by yeasts that live in acidic environments. For this reason, the freeze‐dried samples are dissolved in different standardization buffers according to their pH values. Besides, oxalate buffer is especially recommended for wine samples, in which the presence of transition metal ions (e.g., Fe^3+^, Cu^2+^, Mn^2+^) can compromise stability and reproducibility during NMR measurements. These ions may catalyze oxidation reactions and, more critically, introduce paramagnetic effects that broaden resonances and distort spectral baselines. In this sense, the oxalate buffer acts as a chelating agent and stabilizes the sample matrix. Other considerations that may need to be taken into account include additional steps such as a degassing step—for carbonated or fizzy drinks—or different protocols for non‐water‐soluble liquids or fractions, which should include organic deuterated solvents with their corresponding standard compound, that is, CDCl_3_ with Tetramethylsilane (TMS).
9. It should be noted that the executed commands used for parameter optimization, acquisition, and spectra processing may be named differently according to the commercial spectrometers and software, that is, Varian from Agilent's, JEOL Technics Ltd., etc. However, the physicochemical fundamentals of the technique are the same, and so sequence pulses and parameters can be adapted.
10. After obtaining proper shims (see **step** #**1** from Parameter optimization to perform Nuclear Magnetic Resonance experiments on automated batches), store them in a file using the command *wsh*. Afterwards, during IconNMR acquisition (**step** #**18** from Parameter optimization to perform Nuclear Magnetic Resonance experiments on automated batches), it is possible to program IconNMR to start shimming each sample using the previously optimized shims as reference.
11. When loading sample information into IconNMR Automation Software it is possible to use as the name of all the samples a batch identification name. This way, IconNMR identifies every experiment for each sample using a number (according to the Bruker directory structure). Identifying samples by numbers eases IconNMR Automation, since it is enough to fill out the information for the first sample in its holder row, and after that, copying and pasting into the rest of the holders. When proceeding this way, it is of the utmost importance to keep the information that relates each number coding to its sample.
12. It is recommended to name each spectrum folder sequentially (1, 2, 3, etc.). The executables in TopSpin to convert the spectral information into omics data matrix follow a numerical order. This implies that, if folders are not named sequentially, it is not possible to execute the code for multiple spectra exporting; instead, each spectrum should be exported individually.
13. The commands mentioned for preprocessing and exporting data are also specific to the Bruker TopSpin environment, although these are not unique functionalities of that system. Equivalent functions are readily available in other commercial software, including Mnova (Mestrelab Research) among others.

## Conflict of interest

The authors declare no conflict of interest.

## Author contributions

M. A. R. and I. D.‐M. planned and designed the work. A. F.‐V. and J. H.‐V. analyzed and interpreted the data. A. F.‐V. and J. H.‐V. wrote the manuscript. A. G.‐C., M. A. R., and I. D.‐M. revised and edited the manuscript.

## Data Availability

The spectra used to illustrate the presented method are part of the datasets from previous work [9, 10]. All raw data related to these studies were uploaded to the Metabolomics Workbench repository (https://doi.org/10.21228/M8G82R and https://doi.org/10.21228/M80722) [14], and are available upon request.
